# Differences in changes of data completeness after the implementation of an electronic medical record in three surgical departments of a German hospital–a longitudinal comparative document analysis

**DOI:** 10.1186/s12911-024-02667-0

**Published:** 2024-09-16

**Authors:** Florian Wurster, Christin Herrmann, Marina Beckmann, Natalia Cecon-Stabel, Kerstin Dittmer, Till Hansen, Julia Jaschke, Juliane Köberlein-Neu, Mi-Ran Okumu, Holger Pfaff, Carsten Rusniok, Ute Karbach

**Affiliations:** 1 Faculty of Human Sciences, Faculty of Medicine and University Hospital Cologne, Institute of Medical Sociology, Health Services Research, and Rehabilitation Science, Chair of Quality Development and Evaluation in Rehabilitation, University of Cologne, Eupener Str. 129, 50933 Cologne, Germany; 2 Medical Faculty, Unit of Child Health Services Research, Clinic of General Pediatrics, Neonatology and Pediatric Cardiology, University Hospital Düsseldorf, Heinrich-Heine University Düsseldorf, Moorenstr. 5, 40225 Düsseldorf, Germany; 3 Center for Health Economics and Health Services Research, University of Wuppertal, Rainer-Gruenter-Str. 21, 42119 Wuppertal, Germany

**Keywords:** Electronic medical record, Secondary use, Data quality, Documentation, Completeness, Clinical adoption meta model, Hospital, Health Services research, Implementation, Germany

## Abstract

**Purpose:**

The European health data space promises an efficient environment for research and policy-making. However, this data space is dependent on high data quality. The implementation of electronic medical record systems has a positive impact on data quality, but improvements are not consistent across empirical studies. This study aims to analyze differences in the changes of data quality and to discuss these against distinct stages of the electronic medical record’s adoption process.

**Methods:**

Paper-based and electronic medical records from three surgical departments were compared, assessing changes in data quality after the implementation of an electronic medical record system. Data quality was operationalized as completeness of documentation. Ten information that must be documented in both record types (e.g. vital signs) were coded as 1 if they were documented, otherwise as 0. Chi-Square-Tests were used to compare percentage completeness of these ten information and t-tests to compare mean completeness per record type.

**Results:**

A total of *N* = 659 records were analyzed. Overall, the average completeness improved in the electronic medical record, with a change from 6.02 (*SD* = 1.88) to 7.2 (*SD* = 1.77). At the information level, eight information improved, one deteriorated and one remained unchanged. At the level of departments, changes in data quality show expected differences.

**Conclusion:**

The study provides evidence that improvements in data quality could depend on the process how the electronic medical record is adopted in the affected department. Research is needed to further improve data quality through implementing new electronical medical record systems or updating existing ones.

## Introduction

A digitized healthcare system offers various benefits to all stakeholders involved. There is evidence showing facilitation of working tasks for physicians and nurses [1], the reduction of costs for health care facilities and insurances [2] and an improved quality of care for patients [3]. The corresponding transformation from an analogue to a digital healthcare system has not only changed the way in which the delivery of care or interprofessional collaboration is organized [4]. The increasing digitalization has also a significant impact on how clinical information is assessed and managed. Hospitals are now recording the majority of their documentational tasks through digital platforms such as hospital information systems (HIS) or electronic medical records (EMR). These platforms allow information to be accessible to pre-defined authorized persons independent of time and place [5].

This development has led to an almost exponential growth in the amount of data available in near real time [6]. Even though this data serves the primary purpose of clinical practice and is essential for safety and quality of care [7], the secondary usage of such data sets opens up chances of delivering data-based personalized care [8] and assessing real world data for health services research purposes [9]. This is where the European health data space comes in. As a digital infrastructure, it aims to ensure the secure and interoperable collection of health data from across the European Union. At the patient level, individual disease predictions or optimal treatment options could be determined e.g. with regard to mental and cognitive disorders [10] or oncological care [11]. At the population level, complex causal relationships of pathogenic factors could be identified [12] and issues such as the inadequate care of vulnerable groups be tackled [13]. To be able to do this and avoid possible bias, the underlying datasets must be of high quality.

Data quality is defined as the data’s ability “*to be used effectively*,* economically and rapidly*” [14], or in other words, only “*data that is fit for use*” is sufficient data [15]. According to this wide definition, data quality is a multidimensional construct and there are numerous frameworks, depicting several dimensions of data quality for a wide variety of purposes [16–18]. Exemplary, for the context of health services research, the data’s conformance, completeness and plausibility are named as the most important dimensions of data quality [19], while for the clinical context, the completeness, legibility, accuracy and meaning are postulated as most important [20].

## Completeness of documentation

Of the different dimensions of data quality, completeness can be seen as one of the most important one, since incompleteness is assumed as the most significant threat to data usability [21]. It poses a major risk to utilizing the described data for secondary purposes and leads to uncertainty when planning personalized care or conducting research [22]. An example of this is a decision support system whose functionality was examined with regard to its susceptibility to incomplete data. Run with insufficient completeness scores, the system made incorrect or even unsafe recommendations in up to 77% of the cases [23]. EMRs represent one of the largest sources of the digital data described [24]. It is therefore only consistent that completeness has been the most frequently investigated dimension of data quality regarding the documentation in EMRs since their emergence [22] and is still today [25]. If the concept of completeness is applied to EMRs, various conceptualizations of the completeness of these records emerge [26] (Table 1).

**Table 1:** Conceptualizations of Medical records completeness

Concept	Definition
Documentation Completeness	Completeness is achieved when a record includes all clinical observations that were made during an encounter.
Breadth Completeness	Completeness is achieved when a record includes all relevant data types for secondary use scenarios.
Density Completeness	Completeness is achieved when a record includes a specified number of data points of one information over time.
Predictive Completeness	Completeness is achieved when a record includes sufficient information for training models to predict future events.

However, the reasons for incompleteness of these data are not fully understood yet [27] and explanatory approaches mention a variety of factors, e.g. of social or technical nature [15]. What has been identified in this matter is that the implementation of new EMR systems can have an influence on completeness [28]. The implementation of a new EMR system is even stated as one of the most important factors for increasing completeness [29]. However, improvements in completeness are not consistent across different empirical studies [28].

### Implementation & adoption

This leads to the field of implementation science, in which it is well known that an “one size fits all” approach does not work when implementing digital interventions in healthcare [30]. It is therefore crucial to consider the context in which the implementation takes place [31]. Ignoring the context can even lead to adverse effects of the intervention intended to be beneficial [32] as also described for the implementation of EMR [28], where context can be interpreted e.g. as a technological, financial, cultural or organizational context [33]. However, even within the same context, different changes in data quality are observed [28]. Any differences in changing data quality across studies do therefore not arise exclusively from varying contexts, but could also be influenced by differing adoption processes at the respective departments. In the matter of EMRs, the adoption process is not a linear process with a certain endpoint (as usual). It can be rather seen as an iterative cycle, since EMRs are subject to a constant transformation in order to adapt technological progress (e.g. linking the EMR to a patient portal [34]) or reflect societal demands (e.g. enabling shared clinical notes [35]). This underlines the variety of possibilities to further improve data quality. But to do this, a more comprehensive understanding of influencing factors is required [36].

### Research question

The study presented was part of the overarching *eCoCo* case study, which is described in detail by Beckmann et al. [37]. The main research question of the *eCoCo* case study was about possible effects of the EMR on social aspects of interprofessional collaboration and clinical workflows. The present study represents an independent work package within the *eCoCo* case study with a focus on possible changes in data quality. The completeness of documentation in paper-based medical records (PMR) (pre-intervention) is compared with those in electronic medical records (EMR) (post-intervention) across three surgical departments, aiming to analyze possible differences in the changes of data quality even within a similar context and to discuss possible differences in the changes of data quality against the background of distinct stages of the adoption process.

## Methods

All analyses are based on anonymized data, provided by the departments’ hospital archive, complying with German and European data protection regulations. This includes redacting any patient’s socio-demographics from the records, as well as any information regarding the documenting individual. The *eCoCo* case study was approved by the ethics committee of the medical faculty of the university of cologne (20-1349) and is listed in the German national registry for clinical trials (DRKS00023343).

### Subject of investigation

The study object for the present study was the fever chart document, which is a central part of every inpatient medical record. The fever chart contains basic information about the patient, like the body temperature levels and other vital signs or the experienced pain. The fever chart was selected as specific use case for the study presented because it is considered to be filled out in every inpatient medical record [38]. The hospital is replacing the printed fever chart document by an EMR system (Meona©) in which the fever chart has retained an almost identical format, implying that the information is documented in the same way in both record types (e.g. indicating the pain on a scale of 0–10). Since the various information can be documented in a different frequency, depending on the clinical relevance up to several times a day, the breadth of completeness was chosen as the most appropriate concept of the records completeness. As described, the breadth of completeness means that completeness is achieved when a record includes all relevant data types for secondary use scenarios (Table 1). For the present study’s analysis, all information that has to be recorded as an initial assessment at the day of admission and is documented in both record types was therefore collected for the statistical analysis. This approach means that the completeness of the following ten items was analyzed: *Admission Diagnosis*,* Blood Pressure*,* Body Temperature*,* Code Status*,* Diet*,* Excretions*,* Height*,* Pain*,* Pulse*,* Weight*. This selection mechanism, based on the parallel elements in the record types to be compared, is described as best practice [22] and has been proven in empirical studies [39]. As the data is stored in the archives after the patients have been discharged, any subsequent documentation is consistently assessed in all records, even if the documentation was put in after the day of admission.

### Setting & record sampling

Three surgical departments were included in the study, implying comparable contexts (at least more comparable than comparing surgical with conservative specialities). The three surgical departments included in the study are each representing individual clinics within one large academic teaching hospital in Germany (orthopaedic surgery, cardiac surgery and abdominal surgery). By trying to equate the context, presumed differences in the change in data quality are more likely to be explained by the department-specific adoption processes, rather than organizational context. With regard to the sampling of the analyzed records, records from all three departments were collected, each department contributing both paper-based and electronical records. The records were taken from a three-week period before the implementation process started and a three-week period after the implementation process started, corresponding to all inpatients treated within those periods and departments. German hospitals are required by law to publish publicly available quality reports. Due to the complete anonymization of the individual medical records included, a classic sample description is not possible. The quality reports are therefore used to describe the study sample at department level and include information e.g. on the most frequently treated ICD-codes or the number of beds or working staff. The quality reports describe each department in its entirety on a mean annual basis.

### Data analysis

For each record included, all of the ten items were coded as 1 if they were documented on the day of admission, otherwise they were coded as 0. This resulted in a percentage completeness of each item per record type, e.g. that *blood pressure* was documented in 80% of the paper-based records. In addition, this approach gives a mean completeness per record type, for example that an average of 6 of the 10 items were documented in the electronic records. The percentage completeness of the items in both record types was compared using chi square tests. The mean completeness of the two record types was compared using unpaired t-test. The calculations were made for both the department’s internal pre-post comparison as well as the pooled overall comparison above the three departments. A *P-*value < 0.05 was considered statistically significant for all calculations. The datasets of the three departments were collected in MS Excel and analyzed in SPSS 29 in December 2023.

## Results

### Data base

The completeness of a total of N_total_=659 records was assessed. Of these, the documentation of N_paper_=248 PMRs and N_eletronic_=411 EMRs was analyzed. The detailed distribution of the records by record type, period and department is shown in Table 2.

**Table 2:** Record distribution

	Orthopaedic Surgery		Abdominal Surgery		Cardiac Surgery		Overall	
Record Type	PMR	EMR	PMR	EMR	PMR	EMR	PMR	EMR
**Period**	11/2020	08/2022	05/2019	05/2020	07/2020	07/2021	05/2019–11/2020	05/2020–08/2022
**N**	44	136	95	139	109	136	248	411
**Go Live**	May 2021		June 2019		August 2020		June 2019 – May 2021	

For the departments of cardiac and abdominal surgery, the beginning of the implementation process of the EMR took place right after the period in which the PMRs were collected, with the data collection period for the EMRs being 12 months after the beginning of implementation process. For the department of orthopaedic surgery, the Go Live as well as the second data collection period were delayed due to the COVID-19 pandemic, so that the data collection period for the PMRs was 6 months before the beginning of the implementation process and the data collection period for the EMRs was 15 months after the beginning of the implementation process.

### Setting

Due to the lack of personal data in the analyzed records, the quality reports of the departments are used as a rough description of the study sample. The following information are reported: the departments bed capacity and number of inpatient cases, the total number of physicians and number of nursing staff. Moreover, the top 5 most treated diagnoses (ICD-10) are presented for each department and period.

The numbers of inpatient cases vary from a minimum of *N* = 1787 treated inpatients in the paper-based period on the cardiac department to a maximum of *N* = 2957 treated inpatients in the electronic period on the orthopaedic department, which does not correspond to the departments beds capacity which also peaks in the electronic period on the orthopaedic department (*N* = 97) but shows its minimum in the paper-based period on the abdominal department (*N* = 58). Table 3 offers an overview of assessed characteristics for all three departments per period.

**Table 3:** Departments characteristics

	Orthopaedic Surgery		Abdominal Surgery		Cardiac Surgery	
	Paper 2020	Electronic 2022	Paper 2019	Electronic 2020	Paper 2020	Electronic 2021
**Inpatient cases / year**	2912	2957	1984	1867	1787	1844
**Beds capacity**	94	97	58	62	74	74
**Top 5 Diagnoses (ICD) Treated**	T84, M54, S42, S52, M48	S52, M54, S42, T84, M48	C16, C15, C78, C25, N18	C16, C15, C78, K80, N18	I35, I21, I20, I25, C34	I35, I20, I21, I25, I71
**Number of physicians**	32,06	32,17	23,61	23,10	40,66	44,49
**Number of nursing staff**	74,50	62,21	43,72	51,99	100,99	116,45

### Change of mean completeness

When looking at the mean completeness scores, which describe the average number of documented items out of the total of ten possible items across all records per record type, the t-tests show statistical significance regarding improvements in all three departments, as well as a pooled improvement over all three departments for the EMR. The overall mean score of completeness was 6.02 (*SD* = 1.88) for the PMR type, rising up to 7.2 (*SD* = 1.77) for the EMR type. The lowest mean completeness score was seen in the PMR type on the abdominal department (*m* = 5.91, *SD* = 2.14) which also showed the highest mean completeness score in the EMR type (*m* = 7.3, *SD* = 1.95), implying the highest difference of 1.39. Vice versa the orthopaedical department showed the highest mean completeness score in the PMR type (*m* = 6.25, *SD* = 2.12), but the lowest mean completeness score in the EMR type (*m* = 7.13, *SD* = 2.0), implying the lowest difference of 0.88. All mean completeness scores and differences are stated in detail in Table 4.

**Table 4:** Change of Mean completeness scores per record type

	Orthopaedic Surgery			Abdominal Surgery			Cardiac Surgery			Overall		
Mean Score 0–10	PMR	EMR	*P*	PMR	EMR	*P*	PMR	EMR	*P*	PMR	EMR	*P*
	*N* = 44	*N* = 136		*N* = 95	*N* = 139		*N* = 109	*N* = 136		*N* = 248	*N* = 411	
	6.25	7.13	0.014	5.91	7.30	< 0.001	6.04	7.18	< 0.001	6.02	7.20	< 0.001
**Difference**	0.88			1.39			1.14			1.18		

### Change of percentage completeness

When looking at the changes of percentage completeness of the ten analyzed items, the pooled analysis shows mixed results. Overall, eight of the ten analyzed items exhibited an improvement in the EMR, specifically the documentation of the items *Blood Pressure*,* Body Temperature*,* Code Status*,* Diet*,* Excretions*,* Height*,* Pulse*. Conversely, one item showed a decline (*Admission Diagnosis*) and one item remained unaltered (*Pain*). Differentiated to the three departments, the results show an even more inconsistent picture in some cases. On the one hand, percentage completeness of the item *admission diagnosis* has consistently decreased in all three departments. On the other hand, the items *diet*,* height* and *weight* have consistently increased in all three departments. All other changes do not show the same direction over all three departments. At department level, only three items have improved in orthopaedic surgery, while further three have deteriorated and four remain unchanged, meaning that the overall picture for orthopaedics surgery remains balanced. In abdominal surgery as a counterexample, all items improved, except for the *admission diagnosis* which -as already stated- decreased in all departments and *excretions*, which remained unchanged. This includes that also the documentation of *pain* improved, which deteriorated in the other two departments and remained unchanged at the overall level. All values for the changes of percentage completeness per item are shown in Table 5.

**Table 5:**
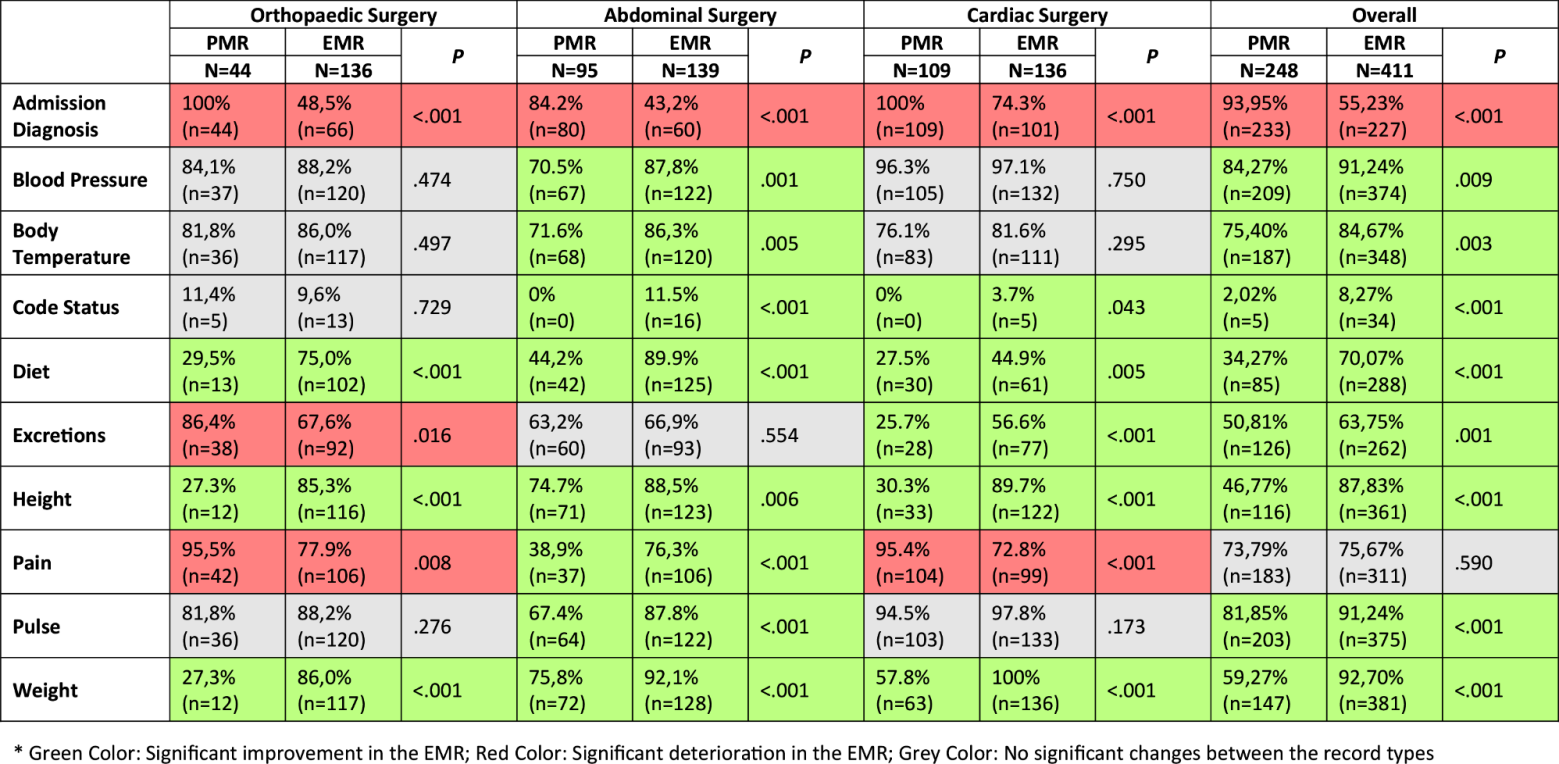
Change of percentage completeness per Item*

## Discussion

The results of the case study show that the implementation of an EMR altered the completeness of documentation within the departments studied. All departments experienced significant improvements in the mean completeness scores, but as expected, with differences across the three departments. In addition, the changes of percentage completeness of the ten individual items show a highly differentiated picture. On the one hand, there are consistent changes of percentage completeness of certain information in all three departments. As an example, percentage completeness of the item *diet* increased in all three departments, while the item *admission diagnosis* decreased in all three departments. On the other hand, there are also some divergent changes with improvements in one department and deterioration in another, like the item *pain* increasing in abdominal surgery, but decreasing in orthopaedic and cardiac surgery. Based on the selection of comparable departments (all surgical disciplines), it can be assumed that the differences in changes are due less to a deviating fundamental context than due to the department-specific adoption process of the EMR.

### Clinical adoption meta model as a frame of reference

**Fig. 1:**
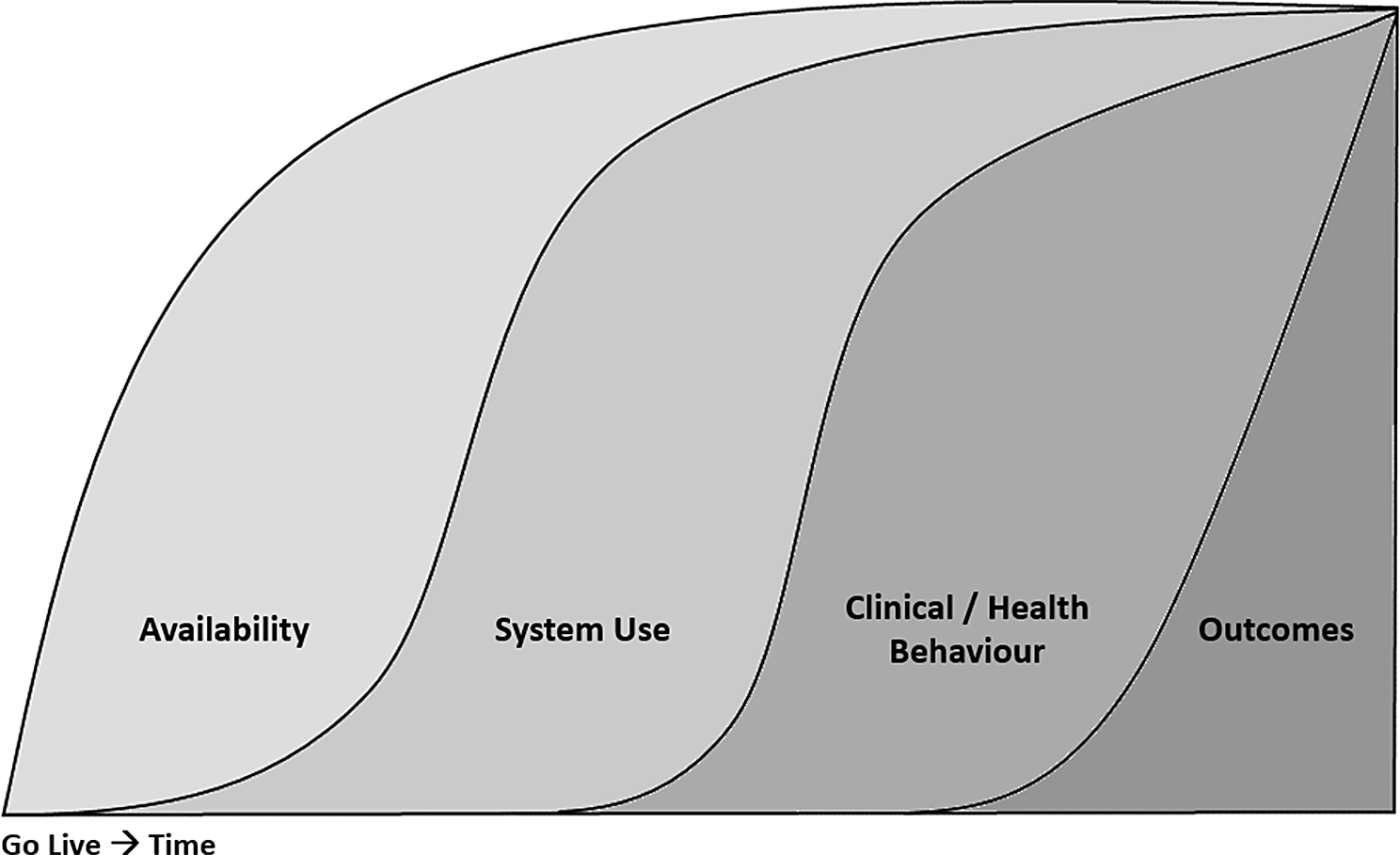
The four dimensions of the clinical adoption meta model (CAMM)

The clinical adoption meta model (CAMM) serves as a framework for categorizing this department-specific adoption process [40]. The CAMM functions as a generic model for describing the adoption process of digital innovations in healthcare [40]. It is made up of the four interrelated dimensions of availability, system use, clinical behavior and outcomes (Fig. 1). In the first step, the EMR must be available. This includes not only the Go Life, but also possible access rights that need to be distributed or mandatory training for end users to be completed. Following the availability, the system use dimension is introduced, which is accompanied by the quantitative usage metrics, such as the number of logins. The third dimension, clinical / health behavior, describes potential changes in behavior or processes and if routines are adjusted to the new EMR. Within the final dimension, potential outcomes like the completeness scores become visible. Due to the comparable context, it is therefore the first three dimensions of CAMM (availability, system use, clinical/health behavior) that are relevant to explain the differences in the changes in completeness across the three departments.

### Availability

In the matter of availability, hardware-related issues are empirically proven which could impede the data entry processes, including poor battery capacity, challenges in docking computer tablets, and malfunctioning touch screens [41], . Moreover, Topaz et al. [42] highlighted a lack of user training, which may weaken data completeness. Although insufficient training or hardware are often cited as a contributor to incomplete documentation, it is noteworthy that both, hospital’s mandatory user training and the hardware used, were consistently the same across the three departments under investigation in this study. The same applies to the EMRs user interface, which can have an influence on the completeness (e.g. with mandatory fields or templates) [43], but is the same across the departments. Therefore, the CAMM’s dimension availability probably plays a subordinate role in explaining the differences in the changes in data quality, shown in this study.

### System use

Similarities across the three departments like the consistent user training, consistent hardware and consistent user interface can also be observed with regard to the CAMM’s dimension system use. On the level of percentage completeness, all three departments have experienced significant declines in the completeness of diagnosis documentation. It should be noted at this point that at the time of the PMR, the diagnosis was entered in an already digital HIS. During the period of the paper-based record, the diagnosis was manually transferred from the digital HIS to the still paper-based pre-print. However, now that both, HIS and EMR are digital, they are run as data silos without an interoperable interface. However, the diagnosis is still being documented in the HIS but is no longer, neither manually nor automatically, transferred into the EMR. This fact makes it clear that both, before and after the beginning of the implementation process of the EMR, a diagnosis was available for each individual patient. Strudwick et al. [44] have already described the same fact that clinical staff experience too heavy workload to document the same information in different places. Here, technical options would be desirable that clearly exist to fill several fields which contain the same information within one system or even across competing systems with just one entry. This is an important point, since although the staff can access the diagnosis in the HIS just as easily as in the EMR, but overall the data quality of the EMR data is more incomplete as a result. However, inadequate system use has consistently led to a deterioration in data quality in all three clinics, so the CAMM’s dimension system use is probably also not explaining the differences in the changes in data quality, shown in this study.

Clinical / Health Behavior.

In view of the fact that the CAMM’s dimensions availability and system use must be regarded as largely the same across the three different departments, the clinical / health behavior appears to have a significant influence. However, this dimension can be subdivided into staff and patients, but with the EMR being only used by staff, solely the aspect of clinical behavior remains relevant. In this matter, clinical behavior can be reflected as the usage of advanced functions of the EMR. An example for such an advanced function would be the connection of intelligent devices to the EMR, like the wireless and automatic transfer of data from blood pressure monitoring systems into the EMR. In this context, there is evidence that due to problems with the wireless transfer, information is still often entered manually and accordingly clinical behavior is not adapted to the advanced or smart options of the EMR [41]. In addition, the staff’s clinical behavior can be strongly influenced by the attributes of the treated patient. In this matter, information could be missing, since they are just not seen as relevant to document. An example is a patient whose diet was not documented. The missing information could also be interpreted that if the diet is not explicitly documented, no special diets need to be considered. This is a known mechanism called informative missingness [45]. The informative missingness mechanism is underlined by the fact that people who are seriously ill have a mean complete documentation above average, because much more information is relevant for those patients and therefore cannot be left blank with the idea of informational missingness [46]. At last, emergencies tend to be less complete documented, logically due to the time pressure under which these patients are treated and their treatment is documented [47]. As clinicians have to decide whether documenting information is worth the time [48], a deep understanding of the importance of complete documentation should be promoted. In this respect, the advanced functions of the EMR should always also be aimed at saving time in the documentation process.

### Limitations

For the presented article, limitations must be stated regarding (1) the interfering impact of the Covid-19 pandemic and (2) the underlying data basis.

(1) As depicted in Table 2, the records distribution cannot be assumed to be equal. Especially the period of the orthopaedic department in which the PMR were collected was affected by the Covid-19 pandemic, when planned interventions were suspended. This might explain the considerable difference between the N_paper_ (*N* = 44) and the N_eletronic_ (*N* = 136) for the orthopaedic department. In terms of the impact of the Covid-19 pandemic on data quality, however, it became apparent that the infectious situation has potentially increased the completeness of the documentation. On the one hand, information was collected as completely as possible in order to avoid additional contact for adding missing information afterwards [49], and also because this information had to contribute to political decision-making in this uncertain situation and complete datasets therefore been requested [50]. However, since the other departments and times were unaffected by the restrictions of the Covid-19 pandemic but also have lower N_paper_ than N_eletronic_, it is conceivable that the PMR type itself has an influence on the number of available records. This is conceivable due to the fact that the PMR could actually not have been found in the department’s archives, whereas the EMRs were of course available for all persons treated. Especially since the number of treated patients remained roughly the same (Table 3), this might explain the differences in analyzed records.

(2) As described in the methods section, the analyses were based on fully de-identified data in accordance with national data protection regulations. As a result, no explicit study sample description was possible, which was countered by describing the annual patient population at department level. Nevertheless, the results can only be interpreted to a limited extent with regard to the possible influence of the actual patients whose treatment was documented, or with regard to the actual person who carried out the documentation. In this regard, there is no information about the comparability of the compared groups of documenting persons (e.g. due to normal staff turnover). In addition, the data only represents the patients from a three-week period. Longer periods could provide more meaningful results here. Future research projects should aim to analyze datasets that contain individual characteristics of the patients and the documenting staff in order to be able to calculate mixed effects models or other more advanced statistical models. In addition, department-specific empirical data at the level of all respective dimensions of the CAMM model could help to better understand the changes in data quality.

## Conclusion & implications

The European health data space and big data analyzability is heavily dependent on sufficient data quality. The completeness of the data is one of the most important dimensions in this regard. This study underlines the importance of implementing or adapting electronic medical records to improve the completeness of this data. At the same time, it strengthens the evidence that the empirically demonstrated improvements might be dependent on the departments’ adoption process. Future research should explicitly address the clinical behavior of the documenting individuals and the technical possibilities to reduce the staff’s documentation burden. Moreover, sufficient datasets should be aimed at to apply more complex statistical models, which should be linked to qualitative data to conclusively understand the dependence on the adoption process. Clinical practice should consider this potential dependency in the inevitable adaptation of their EMRs and conduct the corresponding needs analyses of documenting staff. Furthermore, existing data silos should be dismantled with a focus on interoperability.

## Data Availability

The datasets used and/or analysed during the current study are available from the corresponding author on reasonable request.
